# Prescription Department

**Published:** 1893-10

**Authors:** 


### ANTACID IN PERITONITIS.

By Prof. Jno. E. Clark, Detroit, Mich.

℞ Sodii bicarb. . . . . . . . . . . . . . ʒj.

Acidi tartarici . . . . . . . . . . . ℈ij.

Tr. opii . . . . . . . . . . . . . . gtt ix,

Aq. menth. pip . . . . . . . . . . ℥j.

Aquæ pura, . . . . . . . . . . . . . ℥iij. M

Sig. Teaspoonful every hour or so.

### FOR OBFSITY AND FATTY DEGENERATION OF THE HEART.

℞ Phytoline "Walkers" . . . . . . 2oz.

Sig. Take ten drops six times a day before and after meals in a little water.

### CHRONIC RHINITIS.

In the remedial treatment, the following has proven of service, used with the atomizer twice or thrice daily. If used as a douche, dilute with two or three parts water.

Note: The Iodine is decolorized in preparation, a clear solution of light amber color resulting:

℞ Sodii Boras . . . . . . . . . . . . . ʒss.

Sodii Bicarb . . . . . . . . . . . . ʒi.

Aquæ Puræ . . . . . . . . . . . . ℥ii.

Dissolve and add

Acid Carbol . . . . . . . . . grs. xv.

Tr. Iodi . . . . . . . . . . . . . . . . ʒiii.

Disterine . . . . . . . q. s. ft ℥vi M

EXPECTORATION, TO FACILITATE.

The best remedy to loosen expectoration is muriat of ammonium, combined with some such remedy as syrup of squills. If dry plastic pleurisy be present, iodide of ammonium should be added as follows:

℞. Ammonii muriat..... ʒ iiss.

Syr. scillæ.......... ℥ ij.

Ammonii iodidi...... ʒ iss.

Glycerinæ........... ℥ ss.

Syr. prun.virg. q.s.ad. ℥ iv.

M. Sig.—ʒ j every three hours in water.—*Dr. Anders*,

GONORRHŒA, LATTER STAGES.

℞. Permanganate zinc....gr. iij.

Glycerine ............ ʒ j.

Aquæ distil........... ℥ vj.

M. Sig,—Use an injection three times daily.

A SPECIFIC FOR VOMITING IN PREGNANCY.

℞. Chloroform ........... ʒ ss.

Diovibурnia .......... ℥ ij.

M. Sig. — Dessertspoonful in hot water every hour or two.

ACUTE GASTRO-INTESTINAL TROUBLES OF CHILDREN.

Dr. Sonnenberger recommends:

℞. Resorcin, medicinal....gr. iss–iv.
Infusion of chamomile. ℥ ii ½.
Tincture of rhatany...gr. xxx.
Syr. bitter orange peel.. ℥ v.

A teaspoonful every one or two hours.—*N. Y. Med. Abstract.*

